# DNA repair gene variants are associated with an increased risk of myelodysplastic syndromes in a Czech population

**DOI:** 10.1186/1756-8722-6-9

**Published:** 2013-01-22

**Authors:** Monika Belickova, Michaela Dostalova Merkerova, Eliska Stara, Jitka Vesela, Dana Sponerova, Dana Mikulenkova, Radim Brdicka, Radana Neuwirtova, Anna Jonasova, Jaroslav Cermak

**Affiliations:** 1 Institute of Hematology and Blood Transfusion, U Nemocnice 1, Prague, Czech Republic; 2 First Faculty of Medicine, Charles University, Prague, Czech Republic; 3 First Department of Medicine, General University Hospital, Prague, Czech Republic

**Keywords:** Myelodysplastic syndromes, SNP, DNA repair, Association study

## Abstract

**Background:**

Interactions between genetic variants and risk factors in myelodysplastic syndromes are poorly understood. In this case–control study, we analyzed 1 421 single nucleotide polymorphisms in 408 genes involved in cancer-related pathways in 198 patients and 292 controls.

**Methods:**

The Illumina SNP Cancer Panel was used for genotyping of samples. The chi-squared, p-values, odds ratios and upper and lower limits of the 95% confidence interval were calculated for all the SNPs that passed the quality control filtering.

**Results:**

Gene-based analysis showed nine candidate single nucleotide polymorphisms significantly associated with the disease susceptibility (q-value < 0.05)*.* Four of these polymorphisms were located in oxidative damage/DNA repair genes (*LIG1, RAD52, MSH3* and *GPX3*), which may play important roles in the pathobiology of myelodysplastic syndromes. Two of nine candidate polymorphisms were located in transmembrane transporters (*ABCB1* and *SLC4A2*), contributing to individual variability in drug responses and patient prognoses. Moreover, the variations in the *ROS1* and *STK6* genes were associated with the overall survival of patients.

**Conclusions:**

Our association study identified genetic variants in Czech population that may serve as potential markers for myelodysplastic syndromes.

## Introduction

Myelodysplastic syndromes (MDS) are heterogeneous hematopoietic diseases characterized by ineffective hematopoiesis that frequently transform into acute leukemia. Because genetic background is thought to influence the risk of developing MDS, several case–control studies have investigated the relationships between specific genetic polymorphisms and the risk of MDS [1-5]. For example, the *GSTP1* (glutathione S-transferase pi 1) - 105Val allele has been reported to be associated with an increased risk of MDS [3]. In addition, an association between a polymorphism in the erythropoietin gene and MDS has been described [4]. Despite these studies, the genetic risk factors for MDS remain poorly understood. In this case–control study, we examined single nucleotide polymorphisms (SNPs) in genes associated with an increased risk of MDS in a Czech population.

## Material and methods

### Study population

Peripheral blood or bone marrow samples were obtained from Caucasian Czech patients with *de novo* MDS or acute myeloid leukemia with myelodysplasia-related changes (n = 198) and from Caucasian Czech age and gender matched controls (n = 292) in the Institute of Hematology and Blood Transfusion and the First Department of Internal Medicine, General Faculty Hospital, Prague. Samples were obtained during routine clinical assessment from 2003 to 2010. Only patients with *de novo* MDS without evidence of previous exposure to radiation or chemotherapy were enrolled in the study. The MDS diagnoses were based on the standard diagnostic criteria of the World Health Organization [6]. WHO classification and therapy of patients is summarized in Table 1. All the subjects provided informed consent, and the study was approved by the Local Ethics Committee. The median age of the patients was 63 years (range: 18–89 years), and the median age of the controls was 61 years (range: 19–98 years). Patient group consisted of 69.7% women and control group from 65.4% women.

**Table 1:** Characteristics of patients

**WHO classification**			
	**n/%**	**Blast (%)**	**Cytogenetics, normal/abnormal**
MDS del(5q)	24 (12.1)	2.8	0/24
RCUD	14 (7.1)	1.1	10/2
RARS	12 (6.1)	1.4	5/4
RCMD	59 (29.8)	1.6	36/17
RAEB-1	26 (13.1)	4.4	11/10
RAEB-2	25 (12.6)	12.5	10/9
MDS-U	3 (1.5)	2.0	3/0
MDS/MPS	23 (11.6)	8.3	12/7
AML with MRC	12 (6.1)	28.3	4/6
**Age(yr)**			
Median/range	63 (18–89)		
**Sex**			
Male n/%	60 (30.3)		
Female n/%	138 (69.7)		

MDS del(5q): MDS associated with isolated del(5q**)**; RCUD*:* Refractory cytopenias with unilineage dysplasia; RARS: Refractory anemia with ring sideroblasts; RCMD: Refractory cytopenias with multilineage dysplasia; RAEB-1: Refractory anemia with excess blasts, type 1; RAEB-2: Refractory anemia with excess blasts, type 2; MDS-U: MDS, unclassifiable; MDS/MPS: MDS and myeloproliferative syndromes; AML with MRC: Acute myeloid leukemia with myelodysplasia - related changes.

### Genotyping

We analyzed 1 421 SNPs in 408 genes involved in cancer-related pathways using the Illumina GoldenGate Assay (Illumina Inc., USA). The Cancer SNP Panel contents over 400 genes involved in the etiology of various types of cancer selected from the National Cancer Institute’s Cancer Genome Anatomy Project SNP500Cancer Database. This panel contains more than 3 SNPs, on average, for each gene represented. The list of all tested genes is accessible in Additional file 1: Table S1. The assay was performed according to the manufacturer’s protocol for the Illumina GoldenGate Assay.

### Statistical analysis

The raw data were imported into GenomeStudio V2009.2 (Illumina) for SNP clustering and the generation of genotype calls. The calculations were performed in the statistical programming language R (version 2.12.0; http://www.r-project.org). We excluded 35 samples with an overall call rate < 90% and 15 SNPs with a call rate < 80%. In addition, the deviation of the genotype proportions from Hardy-Weinberg equilibrium (HWE) was assessed in the controls, and 22 SNPs with p-values < 3.56-05 (Bonferroni correction for multiple testing: 0.05/1406) showed significant deviations from HWE and were thus removed. This resulted in the inclusion of a total of 1384 SNPs and 455 samples for our analysis. The chi-squared, p-values, odds ratios (ORs) and upper and lower limits of the 95% confidence interval (CI) of the OR were calculated for all the SNPs that passed the QC filtering. Applying the false discovery rate (FDR) for multiple testing at a 5% significance level according to the Benjamini–Hochberg was supposed to show significant association of the SNP with the phenotype. We identified 9 genes with an adjusted q-value lower than 0.05. Survival plots for the MDS cases were generated using the Kaplan-Meier method, and the differences between the genotypes were assessed using the log-rank test.

### Validation of SNPs results

The results of SNPs status of three selected genes (*LIG1, RAD52, GPX3*) were validated using SNPsTaqMan® Assays (Life Technologies, USA). Paired CD3 cells and CD14 cells were used to distinguish germline or somatic polymorphisms. We compared the SNP status between CD3 T lymphocytes and CD14 monocytes in *LIG1*, *RAD52* and *GPX3* genes in 68 patients.

## Results and discussion

**Figure 1:**
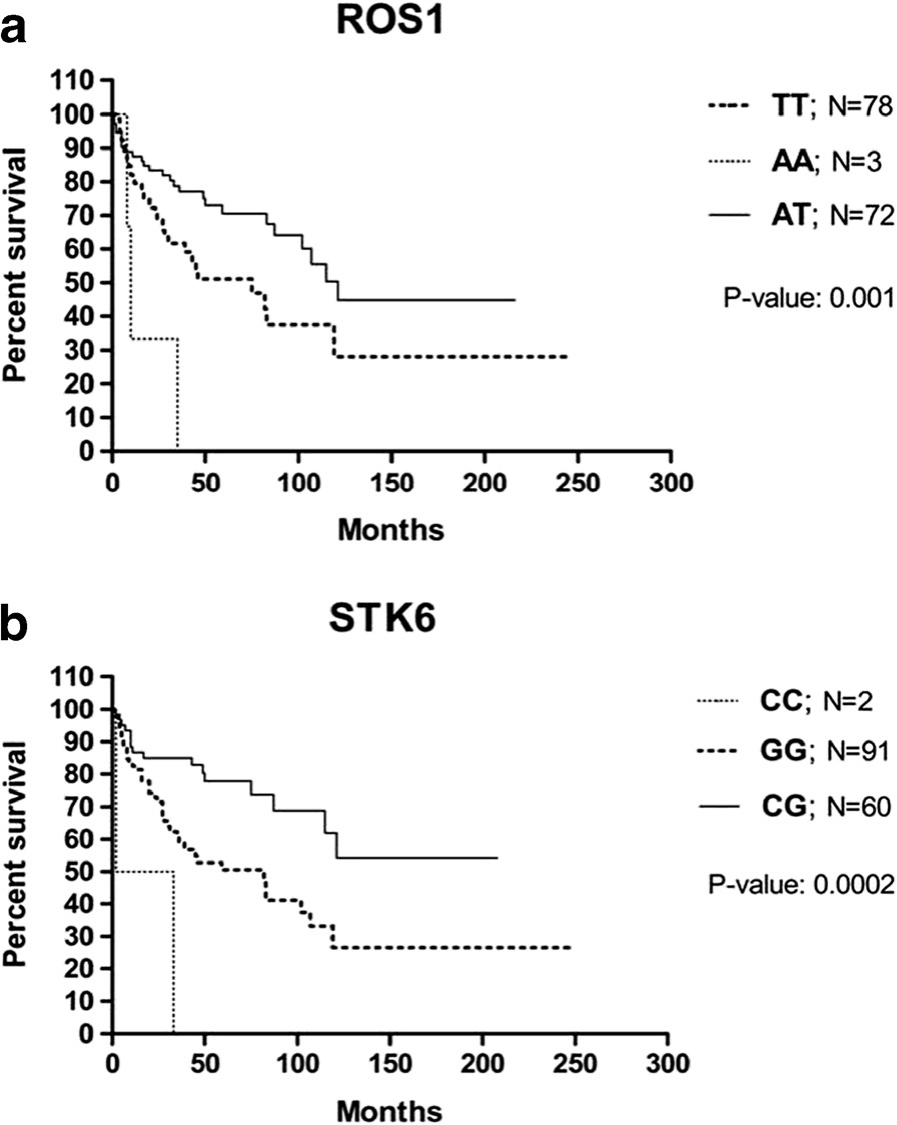
**Survival of myelodysplastic syndrome patients in relation to (1a) *ROS1 *and (1b) *STK6 *genotypes.** The Kaplan-Meier plots show survival (y-axis) versus months from diagnosis (x-axis).

A gene-based analysis identified nine candidate SNPs that were significantly associated (q-value < 0.05) with disease susceptibility. The results of the association analysis are presented in Table 2. The OR of the genes with significant heterozygous and homozygous variants is listed in Table 3. Furthermore, we examined the relationships between the genotyping results and the clinical data. A Kaplan-Maier analysis revealed that alleles in the c-ros oncogene 1 (*ROS1*) gene (p = 0.001) and aurora kinase A (*STK6/AURKA)* gene (p = 0.0002) were independent prognostic factors for survival in our patient cohort (Figure 1). The other genetic polymorphisms identified in this study did not play any prognostic role (*LIG1*: p = 0.11; *ABCB1*: p = 0.08; *SLC4A2*: p = 0.48; *RAD52*: p = 0.41; *PGR*: p = 0.99; *MSH3*: p = NA; *GPX3*: p = 0.64).

**Table 2:** SNPs showing significant genotypic associations with myelodysplastic syndromes

**Gene**	**Name**	**Gene location**	**dbSNPs reference number**	**Gene region**	**Genotype**	**MAF database**	**MAF controls**	**MAF patients**	**OR**	**95% confidence interval**	**P Value**
**SLC4A2**	solute carrier family 4, anion exchanger, member 2	*7q36.1*	rs13240966	intron	C/G	0.227	0.4829	0.7110	2.63	1.94-3.63	7.00E-11
**ABCB1**	ATP-binding cassette, sub-family B (MDR/TAP), member 1	*7q21.12*	rs2235074	intron	A/G	0.031	0.0575	0.1569	3.05	1.88-4.97	8.65E-07
**LIG1**	ligase I, DNA, ATP-dependent	*19q13.2-q13.3*	rs20580	coding	A/C	0.513	0.2216	0.3665	2.03	1.50-2.75	1.65E-06
**ROS1**	c-ros oncogene 1 , receptor tyrosine kinase	*6q22*	rs574664	intron	T/A	0.145	0.1432	0.2593	2.10	1.48-3.00	1.24E-05
**PGR**	progesterone receptor	*11q22-q23*	rs1042838	coding	T/G	0.199	0.1601	0.2804	2.05	1.46-2.87	1.46E-05
**STK6**	aurora kinase A	*20q13*	rs732417	5UTR	C/G	0.083	0.1051	0.1995	2.12	1.43-3.16	7.76E-05
**GPX3**	glutathione peroxidase 3	*5q23*	rs8177426	intron	A/G	0.217	0.2288	0.3567	1.87	1.37-2.55	4.26E-05
**RAD52**	RAD52 homolog	*12p13-p12.2*	rs11226	3UTR	T/C	0.224	0.2308	0.3509	1.80	1.31-2.48	1.70E-04
**MSH3**	mutS homolog 3	*5q11-q12*	rs3797896	intron	G/C	0.066	0.0627	0.0131	0.20	0.07-0.54	2.16E-04

MAF: minor allele frequency; MAF database: Caucasian/European frequencies; OR: odds ratio.

**Table 3:** Association between individual SNPs and MDS

**SNP**	**Genotype**	**Controls N (%)**	**Cases N (%)**	**OR (95% CI)**	**p-Values**
**rs13240966**	***SLC4A2***	235	173		
	**GG**	72 (30.64)	22(12.72)	1.0 (Ref)	
	**CC**	64 (27.23)	95 (57.91)	**4.86** (2.74-8.62)	< 0.0001
	**CG**	99 (42.13)	56 (32.37)	1.85 (1.04-3.30)	0.04
	**CC + CG**	163 (69.36)	151 (87.28)	**3.03** (1.79-5.13)	< 0.0001
**rs2235074**	***ABCB1***	261	188		
	**GG**	232 (88.55)	129 (68.62)	1.0 (Ref)	
	**AA**	1 (0.38)	0 (0.00)	NA	
	**AG**	28 (10.69)	59 (31.38)	**3.79** (2.30-6.24)	< 0.0001
	**AA + AG**	29 (11.07)	59 (31.38)	**3.66** (2.23-6.00)	< 0.0001
**rs20580**	***LIG1***	264	191		
	**CC**	167 (63.26)	84 (43.98)	1.0 (Ref)	
	**AA**	20 (7.58)	33 (17.28)	**3.28** (1.77-6.06)	< 0.0001
	**AC**	77 (29.17)	74(38.74)	**1.91** (1.26-2.89)	0.002
	**AA + AC**	97 (36.74)	110 (57.59)	**2.25** (1.54-3.29)	< 0.0001
**rs574664**	***ROS1***	262	189		
	**TT**	191 (72.90)	95 (50.26)	1.0 (Ref)	
	**AA**	4 (1.53)	4 (2.12)	2.01 (0.49-8.22)	0.32
	**TA**	67 (25.57)	90 (47.62)	**2.70** (1.81-4.03)	< 0.0001
	**AA + TA**	71 (27.10)	94 (49.74)	**2.66** (1.79-3.95)	< 0.0001
**rs732417**	***STK6***	257	188		
	**GG**	203 (78.99)	113 (60.11)	1.0 (Ref)	
	**CC**	0 (0.00)	2 (1.06)	NA	
	**CG**	54 (21.01)	73 (38.83)	**2.43** (1.60-3.70)	< 0.0001
	**CC + CG**	54 (21.01)	75 (39.89)	**2.50** (1.61-3.88)	< 0.0001
**rs1042838**	***PGR***	253	189		
	**GG**	174 (68.77)	86 (45.50)	1.0 (Ref)	
	**TT**	2 (0.79)	3 (1.59)	3.04 (0.40-26.49)	0.34
	**TG**	77 (30.43)	100 (52.91)	**2.63** (1.74-3.97)	< 0.0001
	**TT + TG**	79 (31.23)	103 (54.50)	**2.64** (1.75-3.98)	< 0.0001
**rs8177426**	***GPX3***	260	171		
	**GG**	155 (59.62)	82 (47.95)	1.0 (Ref)	
	**AA**	14 (5.38)	33(19.30)	**4.46** (2.16-9.32)	< 0.0001
	**AG**	91 (35.00)	56 (32.75)	1.16 (0.74-1.82)	0.51
	**AA + AG**	105 (40.38)	92 (53.80)	**1.66** (1.10-2.47)	0.01
**rs11226**	***RAD52***	234	171		
	**CC**	(61.11)	83 (48.54)	1.0 (Ref)	
	**TT**	17 (7.26)	32 (18.71)	**3.24** (1.62-6.53)	0.0002
	**TC**	74 (31.62)	56 (32.75)	1.30 (0.82-2.07)	0.26
	**TT + TC**	91 (38.88)	90 (52.63)	1.70 (1.15-2.54)	0.01
**rs3797896**	***MSH3***	262	191		
	**CC**	232 (88.21)	186 (97.38)	1.0 (Ref)	
	**GG**	3 (1.14)	0 (0.00)	NA	
	**GC**	27 (10.27)	5 (2.62)	**0.23** (0.09-0.61)	0.001
	**GG + GC**	30 (11.40)	5 (2.62)	**0.21** (0.08-0.55)	0.001

The most significant association was observed for the rs13240966 SNP (p = 7.00E^-11^), which is located in the solute carrier family 4, anion exchanger, member 2 (*SLC4A2*) gene. *SLC4A2* is a widely distributed plasma membrane anion exchange protein involved in the regulation of intracellular pH through the exchange of intracellular bicarbonate for extracellular Cl^−^. We detected an association between the homozygous C/C genotype and MDS (OR 4.86; 95% CI 2.74-8.62). This SNP was previously associated with bladder cancer in work by Andrew *et al.*[7]. We also found a significant association between MDS and a polymorphism in a gene that encodes another transmembrane protein, which belongs to the *ABC* (ATP-binding cassette) protein transporter family. *ABC* sub-family B member 1 (*ABCB1*) is responsible for decreased drug accumulation in multidrug-resistant cells and often mediates the development of resistance to anticancer drugs. In our patient cohort, the heterozygous A/G genotype of *ABCB1* was associated with MDS susceptibility (OR 3.79; 95% CI 2.30-6.24). The specific genotype of *ABCB1* has also been proposed to influence the risk of acute lymphoblastic leukemia (ALL), and other *ABCB1* variants may be linked to poor ALL prognoses [8].

The gene that encodes ligase 1 (*LIG1*) was represented in the assay by the marker rs20580. We detected a relationship between the homozygous A/A genotype of this *LIG1* SNP and MDS (p = 1.65E^-06^; OR 3.28; 95% CI 1.77-6.06). *LIG1* plays roles in nucleotide excision repair and in the long-patch base-excision repair pathway. An association of this genetic variant with lung cancer was previously reported by Lee *et al.*[9]. We found significant associations between MDS and of two other polymorphisms in genes related to DNA repair. One of these SNPs was the rs11226 SNP (p = 1.70E^-04^) in the *RAD52* gene, which is involved in DNA double-strand break repair and homologous recombination. The second polymorphism was rs3797896 (p = 2.16E^-04^), which is located in the *MSH3* gene, a component of the post-replicative DNA mismatch repair system. In addition, genetic variants in several other genes involved in DNA repair were significantly associated with MDS (p < 0.01), including rs2238335 in the *BLM* gene (Bloom syndrome, RecQ-helicase-like), rs2308327 in *MGMT* (O-6-methylguanine-DNA methyltransferase), rs4149963 in *EXO 1* (exonuclease 1) and rs7607076 in *MSH2* (mutS homolog-2, colon cancer, nonpolyposis type-1). Previous work has demonstrated that polymorphisms in DNA damage-response genes and DNA repair genes influence DNA repair capacity [10,11]. DNA damage caused by ineffective detoxification or defects in DNA repair can lead to chromosomal instability, which can be associated with tumor formation or progression. Thus, genetic variants in DNA repair may modify the risk of MDS.

We also identified a significant association (p = 4.26E^-05^) between MDS and the rs8177426 SNP in glutathione peroxidase 3 (*GPX3*), with an OR of 4.46 (95% CI 2.16-9.32) for the homozygous A/A genotype. *GPX3* protects cells and enzymes from oxidative damage by catalyzing the reduction of hydrogen peroxide, lipid peroxides and organic hydroperoxide by glutathione. In our previous study, we detected a 1.6-fold decrease in *GPX3* gene expression in MDS patients [12], suggesting that altered gene expression may be an impact of genetic variants.

Another SNP that was significantly associated with MDS in the present work was rs732417 (p = 7.76E^-05^), which is located in the *STK6/AURKA* gene. *AURKA*, a serine/threonine kinase, regulates cell cycle checkpoints and maintains genomic integrity. We detected an association between the heterozygous C/G genotype and the risk of MDS (OR 2.43, 95% CI 1.60-3.70). Allelic variations in *AURKA* have been associated with alterations in its expression level and an increased risk of multiple cancers [13,14]. Andrew *et al.*[7] demonstrated that subjects with certain *AURKA* polymorphisms were significantly more susceptible to bladder cancer. It is noteworthy that the individuals with the homozygous G/G genotype displayed poorer survival than did the patients with other genotypes.

The gene encoding the progesterone receptor (*PGR*) was represented by rs1042838 (p = 1.46E^-05^) and was also found to be associated with MDS. Steroid hormones and their receptors are involved in the regulation of gene expression and can affect cellular proliferation and differentiation in target tissues. *PGR*s signal by binding to other proteins, mainly transcription factors such as *NFKB1*, *AP-1* or *STAT*, which are deregulated in MDS [15,16].

The genotyping analysis also detected an association between rs574664 (p = 1.24E^-05^) in *ROS1* gene and susceptibility to MDS. *ROS1* encodes a proto-oncogene with tyrosine kinase activity that may function as a growth or differentiation factor receptor. Specifically, we detected an association between the heterozygous T/A genotype and MDS (OR 2.70, 95% CI 1.81-4.03). However, the T/T genotype predicted a shorter survival time in the MDS patients in our study.

To date, only limited number of studies [1-5] documented associations with individual SNPs localized particularly in *MTR* (5-methyltetrahydrofolate-homocysteine methyltransferase reductase R)*, RMI1* (RecQ mediated genome instability 1), *GSTP1* (glutathione S-transferase pi 1) and *EPO* (Erythropoietin) genes. However, due to differences in SNPs present on our array that did not include the same polymorphisms as the mentioned studies, we could not confirm these previous results. Recent study of genetic variants of *BLM* (Bloom syndrome, RecQ helicase-like) gene and the proteins that form complexes with *BLM*, such as *TOP3A* and *RMI1*, found the association with cancer risk in acute myeloid leukemia/myelodysplatic syndromes [5]. In our study, we confirmed the association with the *BLM* gene.

Results obtained from the arrays were validated using another genotyping method - SNPsTaqMan® Assays. The correlation coefficient between both methods was 0.99, confirming the microarray results. Additionally, the origin of selected polymorphisms (*LIG1, RAD52, GPX3)* was investigated by comparison of SNP status in paired CD3 monocytes and CD14 T lymphocytes. Genotyping of DNA from separated CD3 and CD14 cells showed the same sequences in both cell lineages, indicating the germline origin of the tested polymorphisms.

## Conclusions

In summary, we identified several genetic variants that may contribute to the pathogenesis of MDS by modi-fying disease risk. Interestingly, five of the polymorphisms described in our study are located close to areas that are frequently deleted in MDS (chromosome 7 - rs13240966 and rs2235074, chromosome 5 - rs8177426 and rs3797896, and chromosome 20 - rs732417). This observation suggests that in addition to large deletions, other types of genetic alterations in MDS-related regions may play a role in the development of MDS. As discussed above, the genetic variants detected in this study are likely to be biologically relevant, particularly the polymorphisms in oxidative damage/DNA repair genes (*LIG1*, *RAD52*, *MSH3* and *GPX3*), which may play important roles in the pathobiology of MDS. Functional polymorphisms in transmembrane transporters (*ABCB1* and *SLC4A2*) may contribute to individual variability in drug responses and patient prognoses. Moreover, the variations in the *ROS1* and *STK6* genes were associated with the overall survival of MDS patients. To our knowledge, this is the first association study to examine the relationships between a large number of SNPs and MDS exclusively in primary MDS patients. Rigorous patient selection is critical because of the differences in etiology observed in primary and secondary MDS, the latter of which is caused by radiation or chemotherapy that is usually administered as a treatment for another type of cancer. Therefore, the incorporation of secondary MDS patients into epidemiological studies could lead to the detection of SNPs related to other types of cancer. This study provides the first evidence of a genetic predisposition to myelodysplasia. However, subsequent genome-wide association studies with larger numbers of subjects are needed to confirm our findings, and further elucidation of the relationships between these genetic variants and MDS is required.

## Findings

Detected genetic variants of DNA repair genes may play important roles in the pathobiology of MDS and polymorphisms in transmembrane transporters may contribute to individual variability in drug responses and patient prognoses.

## Competing interests

The authors declare that they have no competing interests.

## Authors’ contributions

MB and JC designed the research protocol; MB, ES and JV performed investigations, analyzed data; DS, DM, RB, RN, AJ and JC were involved in treating patients, collecting data and helped conceive and design the study; MB and MDM wrote the paper. All authors read and approved the final manuscript.

## Supplementary Material

Additional file 1 **Table S1.** The list of tested genes. (DOC 317 kb) Click here for file

## References

[B1] Kim HN Kim YK Lee IK Yang DH Lee JJ Shin MH Park KS Choi JS Park MR Jo DY Won JH Kwak JY Kim HJ Association between polymorphisms of folate-metabolizing enzymes and hematological malignancies Leuk Res 2009 33 82 87 10.1016/j.leukres.2008.07.026 18774170

[B2] Broberg K Höglund M Gustafsson C Björk J Ingvar C Albin M Olsson H Genetic variant of the human homologous recombination-associated gene RMI1 (S455N) impacts the risk of AML/MDS and malignant melanoma Cancer Lett 2007 258 38 44 10.1016/j.canlet.2007.08.005 17900800

[B3] Fabiani E D’Alò F Scardocci A Greco M Di Ruscio A Criscuolo M Fianchi L Pagano L Hohaus S Leone G Voso MT Polymorphisms of detoxification and DNA repair enzymes in myelodyplastic syndromes Leuk Res 2009 33 1068 1071 10.1016/j.leukres.2008.10.012 19027952

[B4] Ma W Kantarjian H Zhang K Zhang X Wang X Chen C Donahue AC Zhang Z Yeh CH O’Brien S Garcia-Manero G Caporaso N Landgren O Albitar M Significant association between polymorphism of the erythropoietin gene promoter and myelodysplastic syndrome BMC Med Genet 2010 11 163 10.1186/1471-2350-11-163 21078205 PMC2992491

[B5] Broberg K Huynh E Schläwicke Engström K Björk J Albin M Ingvar C Olsson H Höglund M Association between polymorphisms in RMI1, TOP3A, and BLM and risk of cancer, a case–control study BMC Cancer 2009 9 140 10.1186/1471-2407-9-140 19432957 PMC2685436

[B6] Vardiman JW Thiele J Arber DA Brunning RD Borowitz MJ Porwit A Harris NL Le Beau MM Hellström-Lindberg E Tefferi A Bloomfield CD The 2008 revision of the WHO classification of myeloid neoplasms and acute leukemia: rationale and important changes Blood 2009 114 937 951 10.1182/blood-2009-03-209262 19357394

[B7] Andrew AS Gui J Sanderson AC Mason RA Morlock EV Schned AR Kelsey KT Marsit CJ Moore JH Karagas MR Bladder cancer SNP panel predicts susceptibility and survival Hum Genet 2009 125 527 539 10.1007/s00439-009-0645-6 19252927 PMC2763504

[B8] Rao DN Anuradha C Vishnupriya S Sailaja K Surekha D Raghunadharao D Rajappa S Association of an MDR1 gene (C3435T) polymorphism with acute leukemia in India Asian Pac J Cancer Prev 2010 11 1063 1066 21133625

[B9] Lee YC Morgenstern H Greenland S Tashkin DP Papp J Sinsheimer J Cao W Hashibe M You NC Mao JT Cozen W Mack TM Zhang ZF A case–control study of the association of the polymorphisms and haplotypes of DNA ligase I with lung and upper-aerodigestive-tract cancers Int J Cancer 2008 122 1630 1638 18059021 10.1002/ijc.23274 PMC2676936

[B10] Shin A Lee KM Ahn B Park CG Noh SK Park DY Ahn SH Yoo KY Kang D Genotype-phenotype relationship between DNA repair gene genetic polymorphisms and DNA repair capacity Asian Pac J Cancer Prev 2008 9 501 505 18990028

[B11] Beesley J Jordan SJ Spurdle AB Song H Ramus SJ Kjaer SK Hogdall E DiCioccio RA McGuire V Whittemore AS Gayther SA Pharoah PD Webb PM Chenevix-Trench G Australian Ovarian Cancer Study Group, Australian Cancer Study (Ovarian Cancer), Australian Breast Cancer Family Study Association between single-nucleotide polymorphisms in hormone metabolism and DNA repair genes and epithelial ovarian cancer: results from two Australian studies and an additional validation set Cancer Epidemiol Biomarkers Prev 2007 16 2557 2565 10.1158/1055-9965.EPI-07-0542 18086758 PMC2666187

[B12] Vasikova A Belickova M Budinska E Cermak J A distinct expression of various gene subsets in CD34+ cells from patients with early and advanced myelodysplastic syndrome Leuk Res 2010 34 1566 1572 10.1016/j.leukres.2010.02.021 20303173

[B13] Ewart-Toland A Dai Q Gao YT Nagase H Dunlop MG Farrington SM Barnetson RA Anton-Culver H Peel D Ziogas A Lin D Miao X Sun T Ostrander EA Stanford JL Langlois M Chan JM Yuan J Harris CC Bowman ED Clayman GL Lippman SM Lee JJ Zheng W Balmain A Aurora-A/STK15 T + 91A is a general low penetrance cancersusceptibility gene: a meta-analysis of multiple cancer types Carcinogenesis 2005 26 1368 1373 10.1093/carcin/bgi085 15802297

[B14] Matarasso N Bar-Shira A Rozovski U Rosner S Orr-Urtreger A Functional analysis of the Aurora Kinase A Ile31 allelic variant in human prostate Neoplasia 2007 9 707 715 10.1593/neo.07322 17898866 PMC1993855

[B15] Grosjean-Raillard J Adès L Boehrer S Tailler M Fabre C Braun T De Botton S Israel A Fenaux P Kroemer G Flt3 receptor inhibition reduces constitutive NFkappaB activation in high-risk myelodysplastic syndrome and acute myeloid leukemia Apoptosis 2008 13 1148 1161 10.1007/s10495-008-0243-4 18670883

[B16] Davies S Dai D Feldman I Pickett G Leslie KK Identification of a novel mechanism of NF-kappaB inactivation by progesterone through progesterone receptors in Hec50co poorly differentiated endometrial cancer cells: induction of A20 and ABIN-2 Gynecol Oncol 2004 94 463 470 10.1016/j.ygyno.2004.05.028 15297189

