# Supplementary material for: DNA repair gene variants are associated with an increased risk of myelodysplastic syndromes in a Czech population
Source: J Hematol Oncol. 2013 Jan 22;6:9. doi: 10.1186/1756-8722-6-9 (PMC3556100; doi:10.1186/1756-8722-6-9)
Supplement: Additional file 1 — Table S1. The list of tested genes. (DOC 317 kb) [file 1756-8722-6-9-S1.doc]

**Table S1: The list of tested genes.**

| **Gene annotation** | **Chromosome** |
| --- | --- |
| MTHFR | 1 |
| CASP9 | 1 |
| PLA2G2A | 1 |
| HTR1D | 1 |
| OPRD1 | 1 |
| SLC2A1 | 1 |
| AKR1A1 | 1 |
| RAD54L | 1 |
| DIO1 | 1 |
| LEPR | 1 |
| CTH | 1 |
| SEP15 | 1 |
| VCAM1 | 1 |
| GSTM3 | 1 |
| HSD3B2 | 1 |
| HSD3B1 | 1 |
| ARNT | 1 |
| IL6R | 1 |
| CRP | 1 |
| SLAMF1 | 1 |
| APOA2 | 1 |
| RGS5 | 1 |
| SELE | 1 |
| FASLG | 1 |
| RNASEL | 1 |
| NCF2 | 1 |
| PTGS2 | 1 |
| CFH | 1 |
| LMOD1 | 1 |
| IL10 | 1 |
| SLC30A1 | 1 |
| EPHX1 | 1 |
| PARP1 | 1 |
| MTR | 1 |
| EXO1 | 1 |
| APOB | 2 |
| TP53I3 | 2 |
| HADHA | 2 |
| FAM82A | 2 |
| CYP1B1 | 2 |
| ABCG8 | 2 |
| MSH2 | 2 |
| MSH6 | 2 |
| MTHFD2 | 2 |
| IL1A | 2 |
| IL1B | 2 |
| IL1RN | 2 |
| ERCC3 | 2 |
| ABCB11 | 2 |
| PMS1 | 2 |
| STAT1 | 2 |
| CASP10 | 2 |
| CASP8 | 2 |
| ALS2CR12 | 2 |
| FZD7 | 2 |
| CTLA4 | 2 |
| BARD1 | 2 |
| XRCC5 | 2 |
| IGFBP2 | 2 |
| IGFBP5 | 2 |
| IL8RB | 2 |
| IL8RA | 2 |
| IRS1 | 2 |
| UGT1A8 | 2 |
| SEPT2 | 2 |
| OGG1 | 3 |
| PPARG | 3 |
| XPC | 3 |
| MLH1 | 3 |
| CX3CR1 | 3 |
| CTNNB1 | 3 |
| CCR3 | 3 |
| CCR2 | 3 |
| CCR5 | 3 |
| CDC25A | 3 |
| GPX1 | 3 |
| RHOA | 3 |
| TCTA | 3 |
| NICN1 | 3 |
| CD80 | 3 |
| GSK3B | 3 |
| CD86 | 3 |
| CASR | 3 |
| ALDH1L1 | 3 |
| MBD4 | 3 |
| IL12A | 3 |
| MYNN | 3 |
| MASP1 | 3 |
| BCL6 | 3 |
| TP73L | 3 |
| TFRC | 3 |
| SOD3 | 4 |
| GC | 4 |
| IL8 | 4 |
| ADH1C | 4 |
| NFKB1 | 4 |
| EGF | 4 |
| CCNA2 | 4 |
| IL2 | 4 |
| IL15 | 4 |
| FBXW7 | 4 |
| TLR2 | 4 |
| CASP3 | 4 |
| AHRR | 5 |
| SLC6A18 | 5 |
| TERT | 5 |
| SLC6A3 | 5 |
| MTRR | 5 |
| AMACR | 5 |
| IL7R | 5 |
| GHR | 5 |
| SEPP1 | 5 |
| CDK7 | 5 |
| HMGCR | 5 |
| BHMT | 5 |
| DHFR | 5 |
| MSH3 | 5 |
| XRCC4 | 5 |
| CCNH | 5 |
| APC | 5 |
| HSD17B4 | 5 |
| IL3 | 5 |
| CSF2 | 5 |
| IRF1 | 5 |
| IL13 | 5 |
| IL4 | 5 |
| CDC25C | 5 |
| MATR3 | 5 |
| SLC23A1 | 5 |
| DNAJC18 | 5 |
| APBB3 | 5 |
| CD14 | 5 |
| CSF1R | 5 |
| GPX3 | 5 |
| TNIP1 | 5 |
| IL12B | 5 |
| DRD1 | 5 |
| FOXC1 | 6 |
| EDN1 | 6 |
| HFE | 6 |
| LTA | 6 |
| TNF | 6 |
| RXRB | 6 |
| BAK1 | 6 |
| PIM1 | 6 |
| CCND3 | 6 |
| VEGF | 6 |
| NFKBIE | 6 |
| GSTA4 | 6 |
| HTR1B | 6 |
| CGA | 6 |
| ROS1 | 6 |
| ENPP1 | 6 |
| IFNGR1 | 6 |
| ESR1 | 6 |
| RGS17 | 6 |
| OPRM1 | 6 |
| VIL2 | 6 |
| SOD2 | 6 |
| IGF2R | 6 |
| PMS2 | 7 |
| JTV1 | 7 |
| RAC1 | 7 |
| AHR | 7 |
| IL6 | 7 |
| IGFBP1 | 7 |
| IGFBP3 | 7 |
| HUS1 | 7 |
| EGFR | 7 |
| ABCB1 | 7 |
| CALCR | 7 |
| CYP3A7 | 7 |
| CYP3A4 | 7 |
| CAV1 | 7 |
| MET | 7 |
| POT1 | 7 |
| LEP | 7 |
| MEST | 7 |
| NOS3 | 7 |
| CDK5 | 7 |
| SLC4A2 | 7 |
| TNKS | 8 |
| CTSB | 8 |
| MSR1 | 8 |
| LPL | 8 |
| TNFRSF10A | 8 |
| EPHX2 | 8 |
| WRN | 8 |
| POLB | 8 |
| RB1CC1 | 8 |
| GGH | 8 |
| CYP7B1 | 8 |
| TERF1 | 8 |
| NBN | 8 |
| MYC | 8 |
| CDKN2A | 9 |
| NINJ1 | 9 |
| XPA | 9 |
| TGFBR1 | 9 |
| ABCA1 | 9 |
| RAD23B | 9 |
| ALAD | 9 |
| PTGS1 | 9 |
| ENG | 9 |
| RXRA | 9 |
| AKR1C3 | 10 |
| AKR1C4 | 10 |
| IL15RA | 10 |
| FLJ45983 | 10 |
| GATA3 | 10 |
| RET | 10 |
| ALOX5 | 10 |
| ERCC6 | 10 |
| MBL2 | 10 |
| SFTPD | 10 |
| PTEN | 10 |
| FAS | 10 |
| CYP2C19 | 10 |
| ABCC2 | 10 |
| HIF1AN | 10 |
| CYP17A1 | 10 |
| MGMT | 10 |
| CYP2E1 | 10 |
| DRD4 | 11 |
| IGF2 | 11 |
| IGF2AS | 11 |
| CD81 | 11 |
| CDKN1C | 11 |
| SCUBE2 | 11 |
| PTH | 11 |
| TSG101 | 11 |
| LMO2 | 11 |
| CAT | 11 |
| RAG1 | 11 |
| GSTP1 | 11 |
| LRP5 | 11 |
| CCND1 | 11 |
| UCP3 | 11 |
| TYR | 11 |
| PGR | 11 |
| BIRC3 | 11 |
| BIRC2 | 11 |
| MMP1 | 11 |
| NPAT | 11 |
| ATM | 11 |
| ANKK1 | 11 |
| DRD2 | 11 |
| IL10RA | 11 |
| CHEK1 | 11 |
| RAD52 | 12 |
| TNFRSF1A | 12 |
| CD4 | 12 |
| LRP6 | 12 |
| CDKN1B | 12 |
| ARHGDIB | 12 |
| RERG | 12 |
| KRAS | 12 |
| VDR | 12 |
| IGFBP6 | 12 |
| SOAT2 | 12 |
| CDK4 | 12 |
| METTL1 | 12 |
| IFNG | 12 |
| MDM2 | 12 |
| APAF1 | 12 |
| NR1H4 | 12 |
| IGF1 | 12 |
| ALDH2 | 12 |
| HSPB8 | 12 |
| P2RX7 | 12 |
| SCARB1 | 12 |
| PARP4 | 13 |
| BRCA2 | 13 |
| CG018 | 13 |
| ABCC4 | 13 |
| ERCC5 | 13 |
| LIG4 | 13 |
| TEP1 | 14 |
| APEX1 | 14 |
| SLC39A2 | 14 |
| TGM1 | 14 |
| FOXA1 | 14 |
| ESR2 | 14 |
| GPX2 | 14 |
| RAB15 | 14 |
| RGS6 | 14 |
| FOS | 14 |
| GSTZ1 | 14 |
| XRCC3 | 14 |
| AKT1 | 14 |
| OCA2 | 15 |
| PAK6 | 15 |
| RAD51 | 15 |
| SLC30A4 | 15 |
| CYP19A1 | 15 |
| MYO5A | 15 |
| LIPC | 15 |
| CYP1A1 | 15 |
| CTSH | 15 |
| BLM | 15 |
| IGF1R | 15 |
| NUBP2 | 16 |
| IGFALS | 16 |
| LITAF | 16 |
| ERCC4 | 16 |
| PLK1 | 16 |
| IL4R | 16 |
| LOC112869 | 16 |
| CARD15 | 16 |
| CETP | 16 |
| LCAT | 16 |
| CDH1 | 16 |
| TERF2 | 16 |
| NQO1 | 16 |
| HSD17B2 | 16 |
| ZFPM1 | 16 |
| FANCA | 16 |
| ALOX15 | 17 |
| ALOX12 | 17 |
| SLC2A4 | 17 |
| MPDU1 | 17 |
| SAT2 | 17 |
| SHBG | 17 |
| ATP1B2 | 17 |
| TP53 | 17 |
| WDR79 | 17 |
| EFNB3 | 17 |
| NOS2A | 17 |
| LIG3 | 17 |
| CCL5 | 17 |
| ERBB2 | 17 |
| CSF3 | 17 |
| KRT23 | 17 |
| HSD17B1 | 17 |
| BRCA1 | 17 |
| PHB | 17 |
| PCTP | 17 |
| MPO | 17 |
| BRIP1 | 17 |
| AXIN2 | 17 |
| ABCA6 | 17 |
| ABCA5 | 17 |
| TYMS | 18 |
| MBD2 | 18 |
| ABCA7 | 19 |
| GPX4 | 19 |
| STK11 | 19 |
| INSR | 19 |
| PIN1 | 19 |
| ICAM1 | 19 |
| LDLR | 19 |
| JAK3 | 19 |
| GDF15 | 19 |
| MGC20255 | 19 |
| TGFB1 | 19 |
| XRCC1 | 19 |
| ZNF230 | 19 |
| APOE | 19 |
| ERCC2 | 19 |
| PPP1R13L | 19 |
| CD3EAP | 19 |
| ERCC1 | 19 |
| LIG1 | 19 |
| FUT2 | 19 |
| DHDH | 19 |
| BAX | 19 |
| IRF3 | 19 |
| POLD1 | 19 |
| ZNF350 | 19 |
| CDC25B | 20 |
| SLC23A2 | 20 |
| PCNA | 20 |
| BCL2L1 | 20 |
| BPI | 20 |
| MYBL2 | 20 |
| CD40 | 20 |
| NCOA3 | 20 |
| CYP24A1 | 20 |
| STK6 | 20 |
| CSTF1 | 20 |
| MRPL39 | 21 |
| SOD1 | 21 |
| IFNAR2 | 21 |
| IFNGR2 | 21 |
| CBR1 | 21 |
| CBR3 | 21 |
| MX1 | 21 |
| TFF3 | 21 |
| TFF1 | 21 |
| CBS | 21 |
| COL18A1 | 21 |
| SLC19A1 | 21 |
| TXNRD2 | 22 |
| COMT | 22 |
| ARVCF | 22 |
| BCR | 22 |
| XBP1 | 22 |
| SEC14L2 | 22 |
| SSTR3 | 22 |
| PLA2G6 | 22 |
| PDGFB | 22 |
| BZRP | 22 |
| GRPR | X |
| CYBB | X |
| MAOA | X |
| AR | X |
| RPA4 | X |
